# Long‐standing laryngeal rhinoscleroma with rare Mikulicz cells

**DOI:** 10.1002/ccr3.7490

**Published:** 2023-06-07

**Authors:** Raheem Peerani, Manish Shah, Brian Minnema, Hasan Ghaffar, Runjan Chetty, Jan Delabie, Bayardo Perez‐Ordonez, Daniel Xia

**Affiliations:** ^1^ North York General Hospital Toronto Ontario Canada; ^2^ Department of Laboratory Medicine and Pathobiology University of Toronto Toronto Ontario Canada; ^3^ Department of Otolaryngology – Head and Neck Surgery University of Toronto Toronto Ontario Canada; ^4^ Department of Laboratory Medicine St. Michael's Hospital Toronto Ontario Canada; ^5^ Diagnexia Dublin Ireland; ^6^ Division of Hematopathology and Transfusion Medicine University Health Network Toronto Ontario Canada; ^7^ Division of Anatomical Pathology University Health Network Toronto Ontario Canada

**Keywords:** ear, infectious disease, Klebsiella ozaenae, Mikulicz cells, nose and throat, rhinoscleroma

## Abstract

Rhinoscleroma is an infectious granulomatous disease. It is important to identify pathognomonic Mikulicz cells on microscopy, as these can be rare and the chronic inflammatory infiltrate can appear otherwise nonspecific on biopsies.

## Graphical abstract

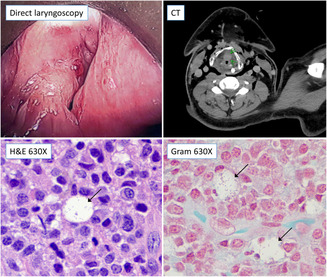

## 1 INTRODUCTION

Rhinoscleroma is a chronic infectious granulomatous disease usually of the nasopharynx and upper respiratory tract, relatively more common in the Middle East and South America, and very rare in nonendemic regions such as North America. Diagnoses are made via histopathology with supporting microbiology cultures. Usual treatment involves long‐term antibiotics; surgery may be necessary if there is airway compromise.

## 2 CASE REPORT

A man in his early 50s presented with worsening stridor, shortness‐of‐breath, and hoarseness over 2 years. Originally from Ecuador, he reported with similar symptoms 7 years ago; workup at that time did not reveal a specific diagnosis; and he was lost to follow‐up.

**FIGURE 1:**
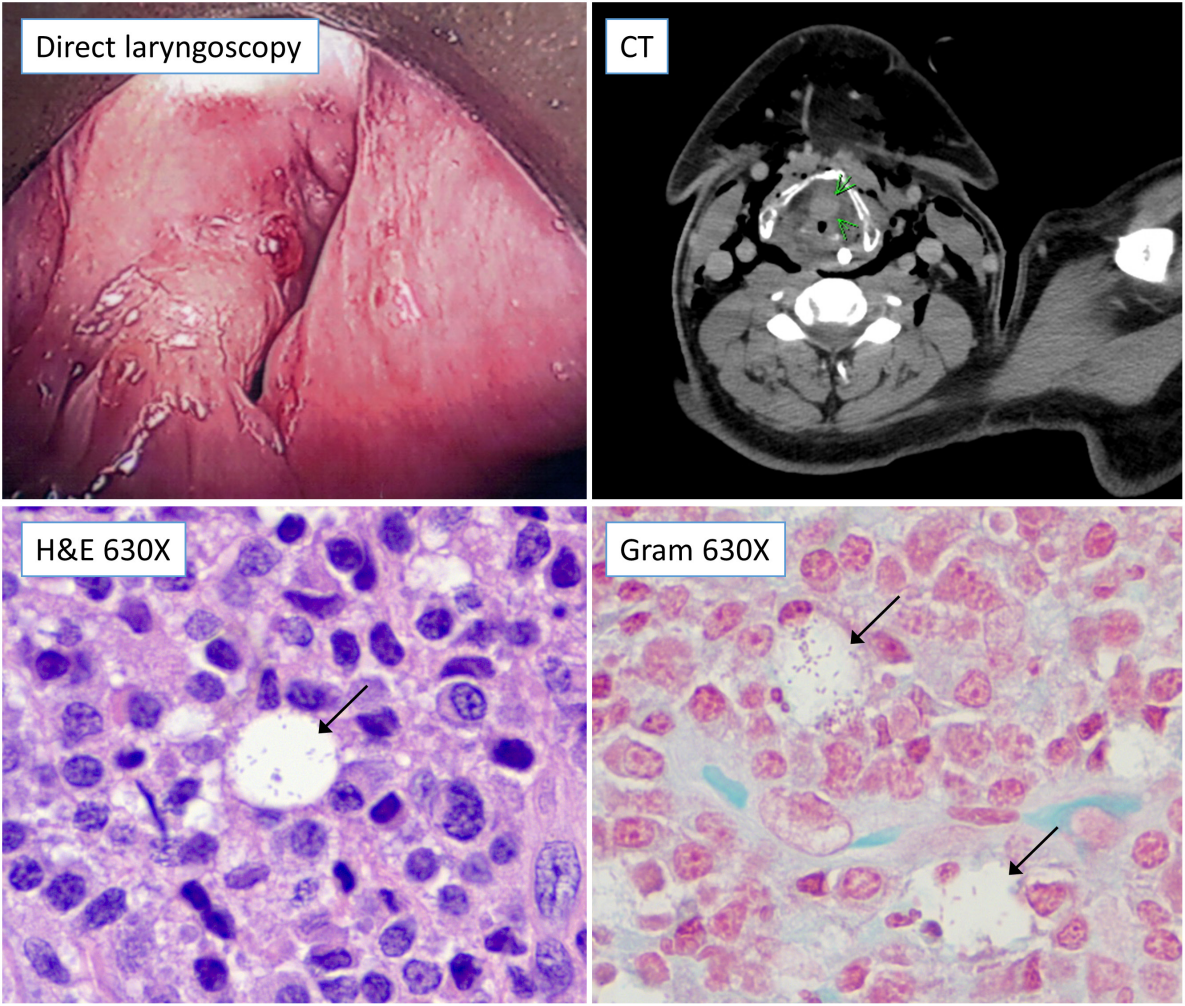
Laryngeal mass. Top left. Direct laryngoscopic image of supraglottic larynx showing an irregular ulcerative mass. Top right. Axial CT images taken after the patient's tracheostomy was completed. There is a mass visible in the supraglottic larynx (green arrows) that is causing narrowing of the airway. Emphysematous changes in the soft tissue are the result of the surgical procedure. Nasogastric tube can be seen in the esophagus. Bottom. On microscopy, H&E (left) and Gram (right) stains show rare Mikulicz cells (black arrows), which are histiocytes that contain intracellular bacteria (gram‐negative rods on the Gram stain), in a background of numerous lymphocytes and plasma cells.

For this presentation, a large obstructing mass on the laryngeal surface of the epiglottis was identified that prevented visualization of the vocal cords and the lower respiratory tract (Figure 1); there was no nasopharyngeal mass. Tracheostomy was performed, and biopsies of the mass were taken. Pathology initially reported a nonspecific dense lymphoplasmacytic infiltrate. Repeat biopsies were performed to clarify the diagnosis.

On further review, both sets of biopsies (Figure 1) showed fragments of squamous mucosa with submucosal fibrosis and a dense chronic inflammatory infiltrate, composed mostly of plasma cells and plasmacytoid lymphocytes. Critically, scattered foamy macrophages, including rare Mikulicz cells with intracellular bacteria (gram‐negative rods on the Gram stain), were identified in the biopsies, suggesting rhinoscleroma.

Confirmatory cultures from the mass showed *Klebsiella ozaenae*. Subsequent ciprofloxacin led to improved stridor, near‐complete regression of the supraglottic mass, with improved vocal cord mobility and patent subglottis; corking trials were carried out with the tracheostomy tube, and the patient was safely decannulated. At 8‐month follow‐up, the laryngeal exam remains stable with no evidence of recurrence/regrowth.

Rhinoscleroma typically affects the nasopharynx and upper respiratory tract; cases involving the larynx^1^, ^2^ and North American cases are rare. While classically associated with *Klebsiella rhinoscleromatis*, other *Klebsiella* species have also been implicated.^3^ This case highlights the challenge of diagnosing rhinoscleroma in its later sclerotic phase, where pathognomonic Mikulicz cells can be rare to absent, and the chronic inflammatory infiltrate may otherwise appear nonspecific on pathology; in this case, the eventual identification of the diagnostic cells led to confirmatory microbiological testing, antibiotic treatment, and good clinical response in a patient with long‐standing disease.

## AUTHOR CONTRIBUTIONS

**Raheem Peerani:** Data curation; investigation; writing – review and editing. **Manish Shah:** Data curation; investigation; writing – review and editing. **Brian Minnema:** Data curation; investigation; writing – review and editing. **Hasan Ghaffar:** Data curation; investigation; writing – review and editing. **Runjan Chetty:** Data curation; investigation; writing – review and editing. **Jan Delabie:** Data curation; investigation; writing – review and editing. **Bayardo Perez‐Ordonez:** Data curation; investigation; writing – review and editing. **Daniel Xia:** Data curation; investigation; writing – original draft; writing – review and editing.

## CONFLICT OF INTEREST STATEMENT

The authors do not have relevant disclosures or conflicts of interest to report.

## CONSENT

Written informed consent was obtained from the patient to publish this report in accordance with the journal's patient consent policy.

## Data Availability

Data sharing is not applicable to this article as no datasets were generated or analysed during the current study.

## References

[1] Umphress B , Raparia K . Rhinoscleroma. Arch Pathol Lab Med. 2018;142:1533‐1536. 30168726 10.5858/arpa.2018-0073-RA

[2] Awad O , Zaky E , Talaat M . Correlation between nasal and laryngeal lesions of Rhinoscleroma in patients of upper Egypt. J Voice. 2020;36:587.e13‐587.e20. doi:10.1016/j.jvoice.2020.07.017 32826120

[3] Gonzales Zamora J , Murali AR . Rhinoscleroma with Pharyngolaryngeal involvement caused by Klebsiella ozaenae. Case Rep Infect Dis. 2016;2016:1‐5. 10.1155/2016/6536275 PMC4880720 27293924

